# Changes in Heart Rate Variability Parameters after Elective Percutaneous Coronary Intervention

**Published:** 2015-04-03

**Authors:** Saeed Abrootan, Saeed Yazdankhah, Babak Payami, Mohammad Alasti

**Affiliations:** 1 *Department of Cardiology, Imam Khomeini Hospital, Ahvaz Jundishapur University of Medical Sciences, Ahvaz, Iran.*; 2 *Department of Cardiology, Bahman General Hospital, Tehran, Iran.*

**Keywords:** Autonomic nervous system, Heart rate, Percutaneous coronary intervention

## Abstract

**Background:** Patients with chronic stable angina often have a state of sympathetic hyperactivity. It is considered associated with myocardial ischemia and disappears after ischemia elimination. The aim of this study was to investigate the changes in heart rate variability parameters, a noninvasive technique for the evaluation of the autonomic nervous system activity, after successful revascularization in these patients to evaluate this theory.

**Methods: **The patients were enrolled among those who underwent successful percutaneous coronary intervention. Short-term heart rate variability analyses of all the patients were obtained, and time-domain indices (standard deviation of normal-to-normal intervals [SDNN], standard deviation of differences of successive R-R intervals [SDSD], root-mean square differences of successive R-R intervals [rMSSD], percentage of R-R intervals differing > 10 ms from the preceding one [PNN_10_], percentage of R-R intervals differing > 20 ms from the preceding one [PNN_20_], percentage of R-R intervals differing > 30 ms from the preceding one [PNN_30_], percentage of R-R intervals differing > 40 ms from the preceding one [PNN_40_], percentage of R-R intervals differing > 50 ms from the preceding one [PNN_50_], percentage of R-R intervals differing > 60 ms from the preceding one [PNN_60_], and percentage of R-R intervals differing > 70 ms from the preceding one [PNN_70_]) were analyzed. All the measurements were made before and after percutaneous coronary intervention.

**Results:** This study included 64 patients, comprising 27 men and 37 women at a mean age of 56.8 ± 9.1 years. There was a significant difference only between pre- and post-revascularization SDNN (27.5 ± 19.72 vs. 41 ± 41.4; p value = 0.013). The other parameters showed no significant differences after successful coronary intervention.

**Conclusion** **:** Our data indicate that the increase in SDNN in patients with stable angina pectoris undergoing percutaneous coronary intervention seems to be prominent.

## Introduction

Heart rate variability (HRV) has been shown to be a noninvasive technique for the evaluation of the activity of the autonomic nervous system.^1^^, ^^2^ Patients with coronary artery disease and exercised-induced angina pectoris have a state of sympathetic hyperactivity, and it seems that the myocardial ischemia is the trigger of this sympathetic hyperactivity.^3^

We hypothesized that the elimination of the trigger factor, i.e. myocardial ischemia, could restore the autonomic balance state to normal. Hence, the aim of the present study was to evaluate the effect that percutaneous coronary intervention (PCI) may have on HRV parameters which indicate the sympathetic-parasympathetic state.

## Methods

The patients were selected from those admitted for elective PCI between 10.1.2012 and 6.30.2013 on the basis of the following inclusion criteria: 1) angiographic evidence of significant stenosis (≥ 70%) in one or two main coronary arteries; 2) stable angina pectoris; 3) successful angiographic PCI of all lesions;^4^ 4) left ventricular ejection fraction ≥ 40%; 5) no history of previous coronary artery bypass grafting (CABG) or PCI; 6) normal electrolyte levels; and 7) normal sinus rhythm.

The exclusion criteria were: 1) need for emergent CABG or repeat PCI during a 24-hour period after the procedure; 2) taking anti-arrhythmic, anti-psychotic, and anti-depressant drugs, which might affect the indices; and 3) twofold increase in cardiac enzymes or sustained monomorphic ventricular tachycardia or ventricular fibrillation during a 24-hour period after PCI.

Atherosclerosis risk factors were defined as follows: Hypertension was defined as taking antihypertensive drugs or baseline blood pressure ≥ 140/90 mmHg. Diabetes Mellitus was defined as taking hypoglycemic agents or fasting plasma glucose level at or above 126 mg/dl, a 2-hour value in an oral glucose tolerance test at or above 200 mg/dl, or a random plasma glucose concentration ≥ 200 mg/dl in the presence of symptoms. Hyperlipidemia was defined as a total cholesterol level ≥ 220 mg/dl or a triglyceride level ≥ 150 mg/dl. Active smoking was defined current use of cigarettes, hookahs, or pipes.^5^

Previous myocardial infarction was defined as the presence of pathologic Q wave on the electrocardiogram (ECG) or imaging evidence of new loss of viable myocardium or new regional wall motion abnormality.^5^

The patients were imaged in the left lateral decubitus position in the parasternal and apical views using a commercially available system (Vingmed Three, General Electric-Vingmed, Milwaukee, WI, USA) during the first day of admission. All the coronary angioplasties were performed at Imam Khomeini General Hospital according to standard techniques. Blood samples were obtained immediately after admission and 24 hours after PCI. Serum creatinine, sodium, potassium, lipid profile, hemoglobin, and cardiac enzymes, including troponin and creatine kinase (CK) concentrations, were measured.

Standard twelve-lead ECG and short-term HRV measurement were done before PCI and 24 hours post-PCI at 7:30 AM using a DMS CardioScan ECG system (DM systems CO., Beijing, China).The evaluation was performed at rest in the supine position for a period of 10 minutes, with the first 5 minutes being used for RR interval stabilization and the subsequent 5 minutes for recording and further analysis. We measured standard deviation of normal-to-normal intervals (SDNN), standard deviation of differences of successive R-R intervals (SDSD), root-mean square differences of successive R-R intervals (rMSSD), percentage of R-R intervals differing > 10 ms from the preceding one (PNN_10_), percentage of R-R intervals differing > 20 ms from the preceding one (PNN_20_), percentage of R-R intervals differing > 30 ms from the preceding one (PNN_30_), percentage of R-R intervals differing > 40 ms from the preceding one (PNN_40_), percentage of R-R intervals differing > 50 ms from the preceding one (PNN_50_), percentage of R-R intervals differing > 60 ms from the preceding one (PNN_60_), and percentage of R-R intervals differing > 70 ms from the preceding one (PNN_70_) as short-term time-domain HRV indices. The measurements of SDNN, SDSD, and rMSSD were reported in milliseconds (ms) and other HRV parameters in percentiles (%). 

The study protocol was approved by the Ethics Committee of Jundishapur University of Medical Sciences. All the patients provided written informed consent.

The continuous data are expressed as mean ± standard deviation. Differences in the mean values before and after the procedure were compared using the paired T-test. A p value < 0.05 was considered statistically significant. For the statistical analyses, the statistical software SPSS version 17.0 for Windows (SPSS Inc., Chicago, IL) was used.

## Results

The study population consisted of 64 patients: 27 (42.2%) men and 37 (57.8%) women at a mean age of 56.8 ± 9.1 years. The baseline characteristics of the study population are summarized in Table 1.

**Table 1:** Baseline characteristics of the patients enrolled in the study (n=64)*

Age (y)	56.8±9.1 (33-85)	Creatinine (mg/dl)	1.1±0.4 (0.5-2.2)
Male/Female	27 (42.2)/37 (57.8)	BUN (mg/dl)	20.9±10.4 (7.9-76)
Weight (kg)	71.9±12.2 (45-110)	Hemoglobin (mg/dl)	12.3±1.9 (7.8-14)
BMI (kg/m^2^)	25.1±4.1 (17-38)	Triglyceride (mg/dl)	150.7±113.1 (45-310)
NYHA class	2.9±0.6	Total cholesterol (mg/dl)	193.6±47.0 (94-366)
Hypertension	27 (42.2)	Sodium (meq/L)	139.3±3.0 (131-148)
Systolic blood pressure (mmHg)	133.0±23.4 (80-200)	Potassium (meq/L)	4.1±0.2 (3.1-5.8)
Diastolic blood pressure (mmHg)	79.1±13.3 (40-120)	LVEF (%)	49.6±4.3 (40-64)
Heart rate (beat/min)	76.2±14.7 (49-140)	Vessels with significant lesions	
Smoking	16 (25.0)	LAD	40 (62.5)
Dyslipidemia	20 (31.3)	LCX	12 (18.8)
Diabetes Mellitus	19 (29.7)	RCA	12 (18.8)
Fasting blood sugar (mg/dl)	131.9±57.2 (76-243)		

BMI, Body mass index; NYHA, New York Heart Association; BUN, Blood Urea Nitrogen; LVEF, Left ventricular ejection fraction; LAD, Left anterior descending artery; LCX, Left circumflex artery; RCA, Right coronary artery

* Data are presented as mean±SD (Max-Min) or n (%)

**Table 2:** Comparison of heart rate variability indices before and after percutaneous coronary intervention*

	Pre-PCI	Post-PCI	P Value
SDNN (ms)	27.5±19.7	41.0±41.4	0.013
SDSD (ms)	30.7±26.2	40.7±41.8	0.103
rMSSD (ms)	30.1±32.1	46.6±58.8	0.056
PNN_10 _(%)	36.3±9.4	35.5±18.2	0.701
PNN_20 _(%)	22.3±15.7	20.8±14.8	0.446
PNN_30 _(%)	11.6±12.2	11.9 ±11.5	0.859
PNN_40 _(%)	6.4±9.6	7.2±9.1	0.543
PNN_50 _(%)	3.5±7.8	5.2±7.8	0.112
PNN_60 _(%)	2.3±7.0	2.0±4.7	0.757
PNN_70 _(%)	2.2±6.3	2.0±4.8	0.801

SDNN, Standard deviation of normal-to-normal intervals; rMSSD, Root-mean square differences of successive R-R intervals; SDSD, Standard deviation of differences of successive R-R intervals; PNN_10_, Percentage of R-R intervals differing > 10 ms from the preceding one; PNN_20_, Percentage of R-R intervals differing > 20 ms from the preceding one; PNN_30_, Percentage of R-R intervals differing > 30 ms from the preceding one; PNN_40_, Percentage of R-R intervals differing > 40 ms from the preceding one; PNN_50_, Percentage of R-R intervals differing > 50 ms from the preceding one; PNN_60_, Percentage of R-R intervals differing > 60 ms from the preceding one; PNN_70_, Percentage of R-R intervals differing > 70 ms from the preceding one

* Data are presented as mean±SD

The patients were on Aspirin (n = 62, 97%), beta blockers (n = 51, 64%), angiotensin-converting enzyme inhibitors (n = 16, 24%), and lipid-lowering medications (n = 21, 33%). Twenty-four patients had significant lesions in two coronary artery territories, and none of our patients had a history of prior myocardial infarction.

The HRV parameters measured before and after PCI are presented in Table 2. 

There was a significant difference only between pre- and post-PCI SDNN, and the other comparisons did not show any significant differences. The p value of the comparison between pre-PCI rMSSD (30.1 ± 32.1 ms) and post-PCI rMSSD (46.6 ± 58.8 ms) was 0.056, which was considerable but insignificant.

We also compared the indices in different groups according to their involved coronary arteries, and there were no significant differences between them.

## Discussion

The major finding of the present study is the significant increase in SDNN, which occurred after successful revascularization in patients with stable angina pectoris. 

In patients with documented coronary artery disease, there is a relationship between the activity of the autonomic system and ischemia. In other words, the nervous system can aggravate ischemia and on the other hand, myocardial ischemia may increase the activity of the autonomic nervous system.^6^ A number of substances released during periods of ischemia can trigger the sympathetic afferent nerves located in the myocardial wall.^7^ There are some ways to assess the cardiovascular autonomic nervous system balance, and the measurement of HRV indices is one of them. The simplicity of this technique is the main reason why many researchers use it to evaluate the activity of the autonomic nervous system in patients with different cardiovascular diseases. 

Pivatelli et al.^8^ found that parasympathetic activity had been reduced in patients with coronary artery disease and that some HRV indices (high frequency [HF] in absolute units, root mean square differences of successive r-r intervals [RMSSD], approximate entropy [ApEn], and total number of adjacent RR intervals with a difference of duration more than 50 ms [NN_50_]) could be used for prognostic purposes in patients with chronic stable angina. Miyase et al.^9^ showed that calculating the low frequency/high frequency (LF/HF) ratio immediately before coronary angiography might predict the presence of coronary artery disease. The results of another study indicated that autonomic heart dysfunction was associated with the severity of coronary occlusion.^10^ Even in asymptomatic subjects without evidence of myocardial ischemia, the severity and extent of coronary atherosclerosis were related to a shift of cardiac autonomic regulation towards sympathetic predominance.^11^

It has been shown that a significant decrease in HRV indices is a predictor of mortality in patients with coronary artery disease.^12^ It is especially true about the patients after myocardial infarction, but can be extended to stable ischemic heart disease. Forslund et al.^6^ found that HRV in the frequency domain had important prognostic value regarding the risk of cardiovascular death in patients with chronic stable angina. They used 24-hour Holter monitoring to calculate frequency domain indices. It was shown in another study that a low HRV was strongly predictive of angiographic coronary disease regardless of other co-morbidities and was clinically useful as a risk predictor in patients with sinus rhythm.^13^

According to the above-mentioned data, the changes in ECG parameters after the elimination of ischemia, including QT dispersion and HRV indices, have been the subject of many researches.

Increased QT dispersion reflects inhomogeneous ventricular repolarization, which can provide a background for clinically important ventricular arrhythmias and the shortening of QT dispersion after successful revascularization has been shown in some previous studies.^14^^, ^^15^ Gomes et al.^3^ found that cardiac revascularization reduced central sympathetic activity in patients with myocardial ischemia. They used a two-channel Holter to calculate the mean LF/HF ratio as a frequency domain HRV parameter during a 10-minute period and showed that the ratio decreased after revascularization. Erdogan et al.^12^ showed that revascularization in the patients with chronic total occlusion decreased time- and frequency-domain HRV parameters but none of them reached statistical significance. They speculated that the decrease in HRV parameters in early periods following the recanalization of chronic totally occluded arteries might be associated with acute endothelial injury or micro-embolization to the vascular bed. Also, the localization of totally occluded coronary arteries did not have significant impact on the results. Aydinlar et al.^16^ in another study found that the components of HF, rMSSD, and SDNN were increased after PCI significantly. They used the Holter monitoring system to calculate short-term HRV parameters before and after balloon inflation. We used an ECG system instead of a Holter monitoring system to measure short-term time-domain HRV parameters and calculated these parameters during a 5-minute period. We found that SDNN increased significantly in patients with stable angina pectoris after successful revascularization. We also observed that rMSSD increased after PCI, but the difference did not reach statistical significance. The reason might be the low number of our patients. The other time-domain parameters did not change significantly.

Our study has some important limitations. The major limitation of the present study may be represented by the small number of the patients. As a result, designing a larger study with more cases could be more informative. The other limitation is that we only used time-domain HRV parameters. 

## Conclusion

In conclusion, our data indicate that among different time-domain HRV parameters, only SDNN increases significantly after successful revascularization in patients with stable angina pectoris. 

## Notes:

***This paper should be cited as:*** Abrootan S, Yazdankhah S, Payami B, Alasti M. Changes in Heart Rate Variability Parameters after Elective Percutaneous Coronary Intervention. J Teh Univ Heart Ctr 2015;10(2):80-84.
